# Soft-tissue sarcoma with MN1–BEND2 fusion: second reported case and comparative analysis

**DOI:** 10.1093/jscr/rjag481

**Published:** 2026-06-27

**Authors:** Nicole Nagib, Abanoub Gabra, David Joyce, Rachel Voss, David Becker-Weidman, Marilyn Bui

**Affiliations:** Moffitt Cancer Center, Tampa, FL 33612, United States; Department of Pathology, Moffitt Cancer Center, Tampa, FL 33612, United States; Department of Surgery, Moffitt Cancer Center, Tampa, FL 33612, United States; Department of Surgery, Moffitt Cancer Center, Tampa, FL 33612, United States; Department of Radiology, Moffitt Cancer Center, Tampa, FL 33612, United States; Department of Pathology, Moffitt Cancer Center, Tampa, FL 33612, United States

**Keywords:** astroblastoma, sarcoma, retroperitoneum

## Abstract

MN1–BEND2 fusion is a defining molecular alteration in astroblastoma, MN1-altered, and is rarely identified in extracranial tumors. To date, only one soft-tissue sarcoma harboring this fusion has been reported. We present the second known case, arising in the deep pelvis of a 34-year-old woman with osseous and neural foraminal invasion. Imaging demonstrated a destructive pelvic mass, and biopsy revealed a spindle cell sarcoma with a myofibroblastic immunophenotype. The patient underwent neoadjuvant radiotherapy followed by radical resection. Histologic evaluation showed heterogeneous morphology, and molecular testing confirmed MN1–BEND2 fusion. The patient remains without evidence of recurrence on short-term follow-up. This case expands the clinicopathologic spectrum of MN1–BEND2–associated soft-tissue sarcomas and highlights the importance of molecular testing in undifferentiated spindle-cell tumors to ensure accurate diagnosis and classification.

## Introduction

The MN1–BEND2 fusion is a defining molecular alteration in astroblastoma, MN1-altered, a tumor entity recognized in the 5th edition of the World Health Organization classification of central nervous system tumors [1]. Outside the central nervous system, this fusion is exceedingly rare. To date, only a single soft-tissue sarcoma harboring MN1–BEND2 has been reported [2].

MN1 rearrangements have been identified across a spectrum of tumors and are associated with diverse morphologic and molecular profiles, including distinct methylation-based classifications in central nervous system neoplasms [3–6]. However, their occurrence in extracranial mesenchymal tumors remains poorly characterized.

We report the second known case of MN1–BEND2 soft-tissue sarcoma, arising in the deep pelvis with osseous and neural foraminal invasion, and compare its clinicopathologic and molecular features with the previously reported case to further define this emerging entity.

## Case presentation

A 34-year-old woman with a history of polycystic ovarian syndrome and prior pelvic teratoma resection presented after an incidental posterior pelvic mass was identified on ultrasound during evaluation for irregular menses. The patient had undergone teratoma resection ~8 years prior; however, slides from the prior specimen were unavailable for review. She reported a 6-month history of intermittent left-sided pelvic pain, previously limiting weight-bearing, without radicular symptoms at presentation.

**Figure 1:**
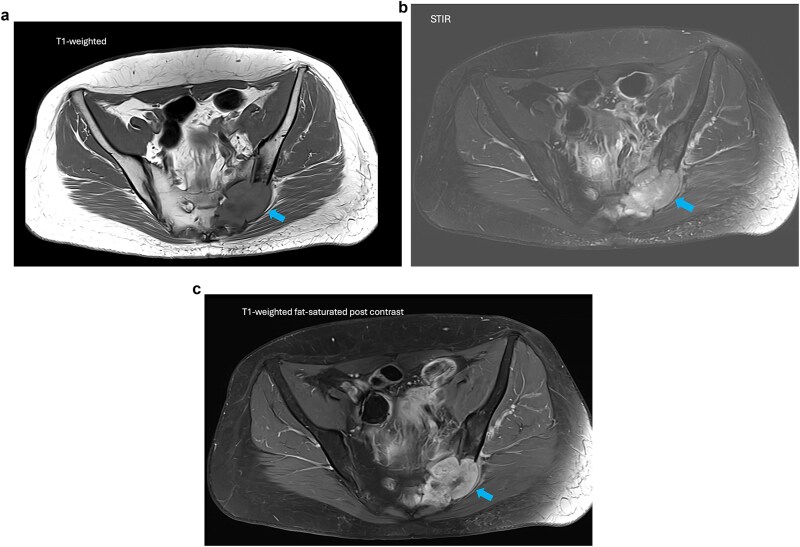
MRI of the pelvis. (a) Axial T1-weighted image demonstrating a lobulated low-signal mass centered in the left sacrum. (b) Axial STIR sequence showing heterogeneous high signal intensity with osseous destruction and soft tissue extension. (c) Axial T1-weighted fat-saturated post-contrast image demonstrating heterogeneous enhancement with small internal foci of necrosis.

Magnetic resonance imaging (MRI) and computed tomography (CT) demonstrated a lobulated, heterogeneously enhancing mass centered in the left posterior pelvis measuring up to 8.9 cm, involving the sacrum (S1–S4), sacroiliac joint, iliac bone, and piriformis muscle (Fig. 1). Imaging showed osseous destruction, pathologic fracture lines, and extension into the neural foramina. CT imaging of the chest, abdomen, and pelvis demonstrated no evidence of distant metastatic disease.

Core needle biopsy revealed a spindle cell sarcoma with scattered multinucleated giant cells and minimal necrosis (Fig. 4). Immunohistochemistry showed positivity for vimentin and smooth muscle actin, with negative staining for S100, desmin, cytokeratin, STAT6, and MUC4.

**Figure 2:**
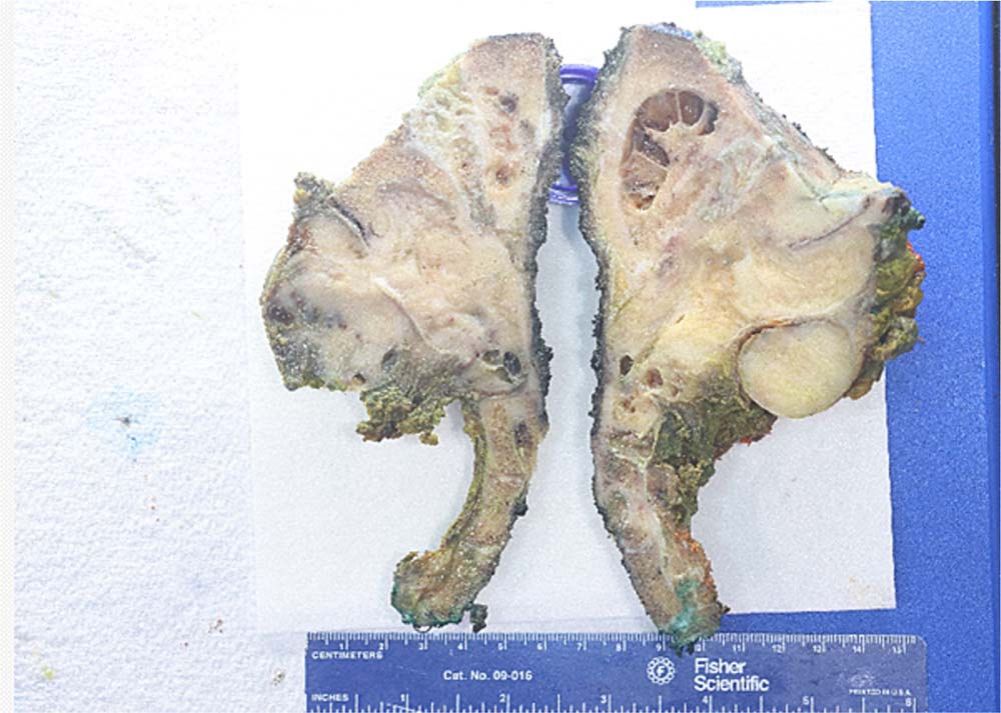
Gross resection specimen from left hemipelvectomy. The bisected specimen demonstrates a firm tan-white mass involving the sacral and iliac bone with extension into adjacent soft tissue. Areas of cystic change and hemorrhage are present. Scale shown in centimeters.

**Figure 3:**
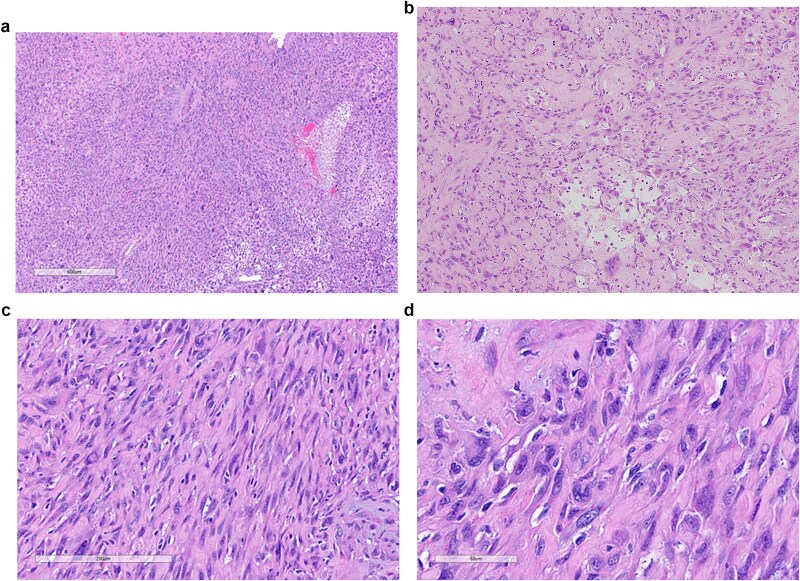
Histopathologic features of the resection specimen. (a) Low-power view (H&E, 4×) demonstrating diffuse infiltration of sarcomatous tissue. (b) Intermediate magnification (H&E, 10×) showing spindle-cell proliferation within collagenous stroma. (c) Higher magnification (H&E, 20×) demonstrating pleomorphic spindle cells with scattered multinucleated giant cells. (d) High-power view (H&E, 40×) illustrating high-grade nuclear atypia and low mitotic activity.

The patient underwent neoadjuvant intensity-modulated radiotherapy followed by radical resection with left hemipelvectomy. Gross examination demonstrated a destructive mass involving the sacrum and adjacent soft tissue (Fig. 2). Histologic evaluation of the resection specimen showed heterogeneous morphology with low- and high-grade components, including pleomorphic spindle cells, multinucleated giant cells, tumor necrosis, and stromal fibrosis (Fig. 3). Immunohistochemistry remained negative for lineage-specific markers, with a Ki-67 proliferation index of ~15% and p53 immunostaining interpreted as mutant-type expression (Fig. 5). Next-generation sequencing confirmed an MN1–BEND2 fusion. Molecular testing was performed using the Moffitt Solid Tumor Fusion eXtended Panel (SafireX), based on the Archer FusionPlex Pan Solid Tumor v2 platform with coverage of 137 genes.

**Figure 4:**
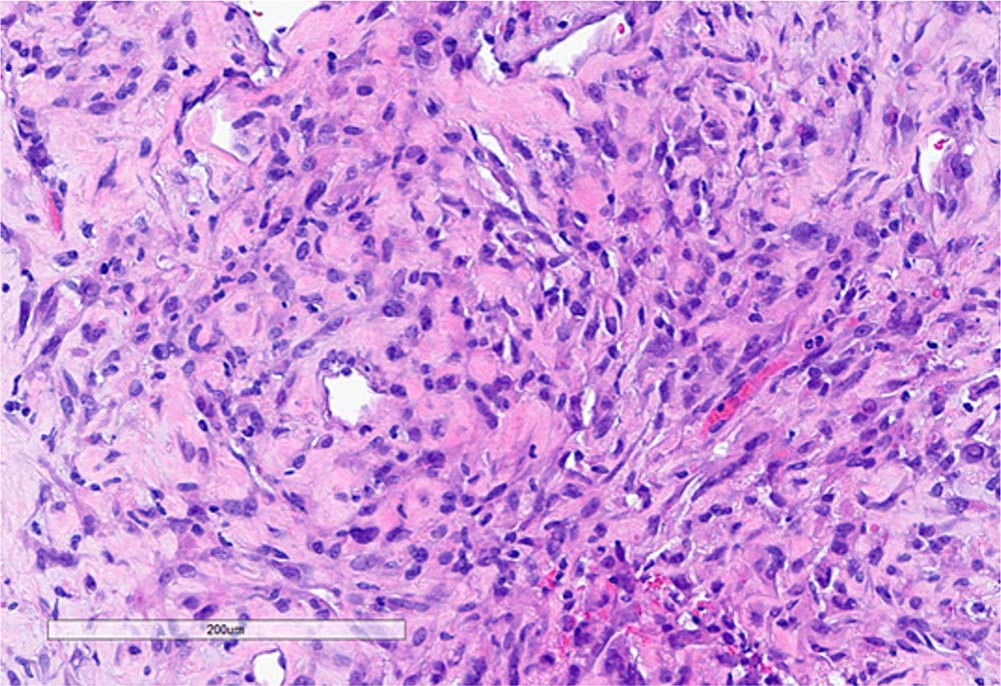
Core needle biopsy specimen. (a) Spindle cell neoplasm composed of atypical spindle cells embedded in fibrous stroma (H&E, 20×).

**Figure 5:**
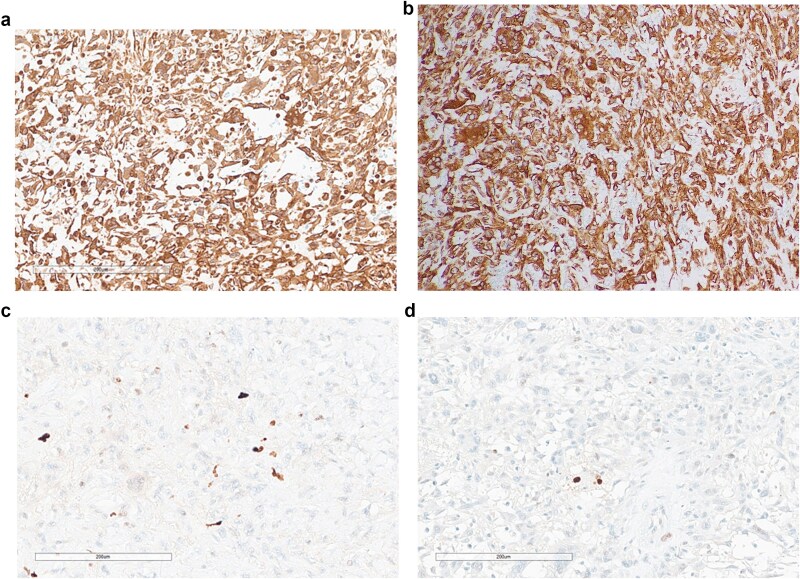
Immunohistochemical findings. (a) Smooth muscle actin (SMA) positivity. (b) Diffuse vimentin positivity. (c) Ki-67 proliferation index ~15%. (d) p53 immunostaining interpreted as mutant-type expression (null pattern).

Postoperatively, the patient recovered well, with residual neuropathic pain managed medically. Surveillance imaging has shown no recurrence after 9 months of follow-up.

## Discussion

MN1–BEND2–associated neoplasms are rare, with only a single soft-tissue sarcoma reported prior to this case [2]. Our findings expand the anatomic and clinicopathologic spectrum of these tumors, demonstrating that this fusion can occur in deep pelvic soft tissues with osseous and neural involvement. Key clinicopathologic features of both cases are summarized in Table 1.

**Table 1:** Comparison of MN1–BEND2 soft-tissue sarcomas.

**Feature**	**Present case**	**Yoshida *et al.*** [2]
Age / Sex	34-year-old female	87-year-old female
Location	Deep pelvis	Abdominal wall
Tumor Size	~8.9 cm	Not specified
Invasion	Osseous + neural foramina	Local soft tissue
Morphology	Spindle cell with pleomorphism and multinucleated giant cells	Biphasic: low-grade spindle + high-grade oval cells
Mitotic activity	Low	Low
Necrosis	Present (partly post-treatment)	Limited
SMA	Positive	Positive
S100	Negative	Negative
Desmin	Negative	Negative
Cytokeratin	Negative	Negative
STAT6 / MUC4	Negative	Negative
Ki-67	~15%	~5%
Molecular Finding	MN1–BEND2 fusion	MN1–BEND2 fusion
Treatment	Neoadjuvant RT + surgical resection	Surgical resection
Outcome	No recurrence (short-term follow-up)	No recurrence (short-term follow-up)

Compared with the previously reported case by Yoshida *et al.*, both tumors were deep-seated and driven by MN1–BEND2 fusion, supporting the possibility of a shared molecular oncogenic mechanism across anatomically distinct sites [2]. Notably, our case occurred in a substantially younger patient and demonstrated more extensive local invasion, including osseous destruction and neural foraminal involvement, suggesting potential variability in clinical presentation and behavior.

The biologic role of MN1–BEND2 fusion remains incompletely understood; however, MN1 rearrangements are thought to function as oncogenic transcriptional drivers and have been implicated in distinct molecular tumor subgroups, particularly within central nervous system neoplasms [1, 3, 4]. BEND2 encodes a BEN-domain containing protein involved in chromatin organization and transcriptional regulation, and fusion with MN1 may contribute to aberrant transcriptional activity and tumorigenesis [3, 4]. The identification of this fusion in both astroblastoma, MN1-altered, and rare extracranial sarcomas raises the possibility of a shared molecular oncogenic mechanism across anatomically distinct neoplasms [1, 2].

Recognition of MN1–BEND2 sarcoma is diagnostically important, as its morphologic and immunohistochemical features overlap with entities such as low-grade myofibroblastic sarcoma, malignant peripheral nerve sheath tumor, and undifferentiated pleomorphic sarcoma. In such cases, the absence of lineage-specific markers should prompt molecular testing. As demonstrated here, RNA-based sequencing was essential for establishing the diagnosis.

Although limited by the small number of reported cases, both patients have demonstrated no evidence of recurrence on short-term follow-up. Our case further suggests that multimodal therapy, including radiotherapy and surgical resection, may achieve local disease control. Additional cases and longer follow-up are required to better define the biologic behavior and prognosis of MN1–BEND2–associated soft-tissue sarcomas.

## Conclusion

MN1–BEND2 soft-tissue sarcoma represents an emerging and rare entity, with this case representing the second reported occurrence. Our findings expand the anatomic and clinical spectrum of these tumors and highlight the importance of molecular testing for accurate diagnosis in undifferentiated spindle-cell sarcomas. Further case accumulation and follow-up are needed to better define their behavior and prognosis.
